# Intramolecular Coupling of Terminal Alkynes by Atom Manipulation

**DOI:** 10.1002/anie.202009200

**Published:** 2020-10-13

**Authors:** Florian Albrecht, Dulce Rey, Shadi Fatayer, Fabian Schulz, Dolores Pérez, Diego Peña, Leo Gross

**Affiliations:** ^1^ IBM Research–Zurich 8803 Rüschlikon Switzerland; ^2^ Centro de Investigación en Química Biolóxica e Materiais, Moleculares (CiQUS) and Departamento de Química Orgánica Universidade de Santiago de Compostela 15782 Santiago de Compostela Spain

**Keywords:** atomic force microscopy, atom manipulation, C−C coupling, on-surface synthesis, scanning tunneling microscopy

## Abstract

Glaser‐like coupling of terminal alkynes by thermal activation is extensively used in on‐surface chemistry. Here we demonstrate an intramolecular version of this reaction performed by atom manipulation. We used voltage pulses from the tip to trigger a Glaser‐like coupling between terminal alkyne carbons within a custom‐synthesized precursor molecule adsorbed on bilayer NaCl on Cu(111). Different conformations of the precursor molecule and the product were characterized by molecular structure elucidation with atomic force microscopy and orbital density mapping with scanning tunneling microscopy, accompanied by density functional theory calculations. We revealed partially dehydrogenated intermediates, providing insight into the reaction pathway.

## Graphical abstract

We demonstrated a Glaser‐like coupling reaction by atom manipulation. The intramolecular reaction was performed within a single molecule, facilitated by the design of the precursor molecule. By using high‐resolution atomic force microscopy, we revealed precursors, intermediates, and products. Resolving and manipulating the positions of individual hydrogen atoms in intermediates provided information about the reaction pathway.

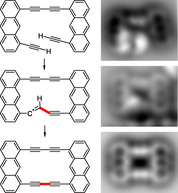

On‐surface synthesis has made great progress since the demonstration of covalent coupling by thermal activation pioneered by Grill et al. in 2007.^[1]^ Milestones include on‐surface‐created polymers,^[2]^ graphene nanoribbons,^[3]^ a plethora of novel molecules and a wealth of one‐ and two‐dimensional nanostructures with intriguing properties.[^4^, ^5^] By contrast, the on‐surface covalent coupling by atom manipulation is still in its infancy, and only a few successful examples have been reported so far.[^6^, ^7^, ^8^, ^9^]

On‐surface chemistry by thermal activation, on the one hand, and by atomic manipulation, on the other hand, are complementary approaches with different advantages and challenges. In general, large defect‐free structures with identical repeating units can be produced by thermal activation. With atomic manipulation, every single reaction step can be induced and studied separately,^[8]^ defects can be deliberately introduced, units do not need to repeat, and thus more complex custom designed structures far from equilibrium could be fabricated in the future.^[10]^ In addition to its flexibility, atom manipulation has the advantage that often reactions do not require the catalytic activity of the metallic substrate and can be performed on insulating films. On ultrathin insulating films, for example, few layer NaCl or monolayer Xe, also reactive intermediates can be stabilized[^8^, ^11^] and structures are electronically decoupled, which is advantageous for their electronic characterization^[12]^ and possible molecular electronics applications.

The oxidative coupling of terminal alkynes promoted by cuprous salts is a well‐known and classic reaction in organic synthesis, frequently named as the Glaser‐Hay coupling (Scheme 1 a).^[13]^ Among the reactions successfully introduced in the field of on‐surface synthesis, this coupling reaction occupies a prominent position. It was employed for building polymers on metallic surfaces[^14^, ^15^, ^16^, ^17^, ^18^, ^19^, ^20^, ^21^] and recently was also observed on the surface of a bulk insulator by thermal activation.^[22]^ However, this reaction has never been demonstrated by atom manipulation up to now. Here we report the first example of on‐surface Glaser coupling by atom manipulation on single molecules.

**Scheme 1:**
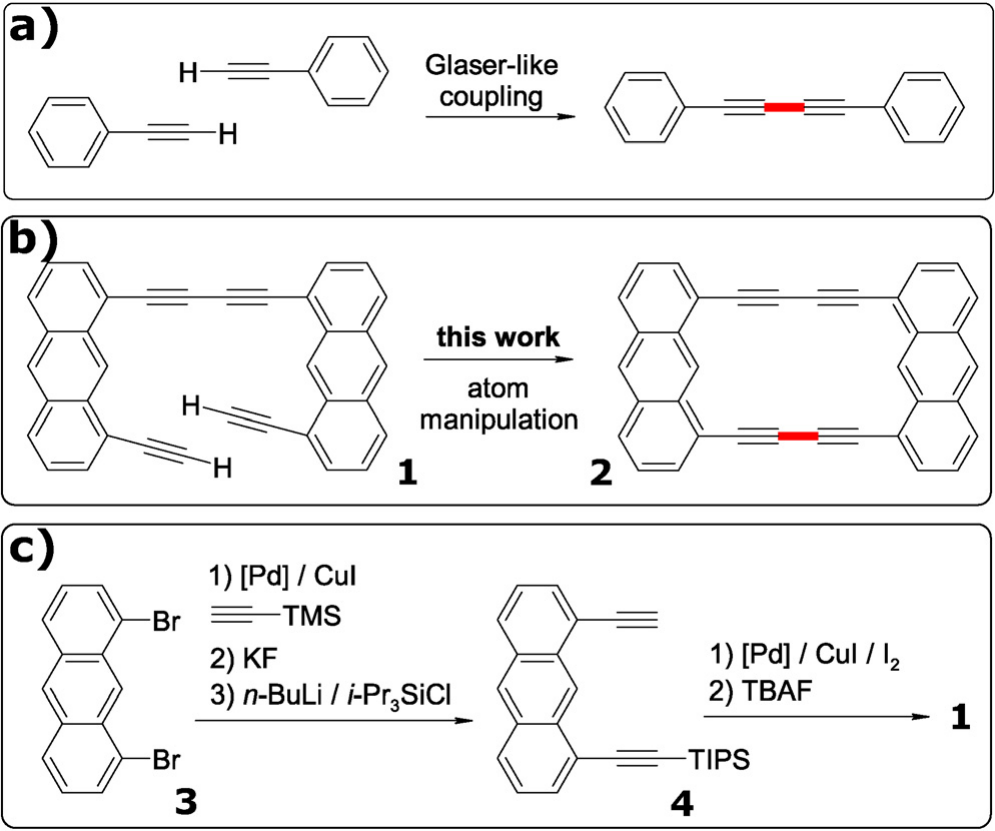
a) Intermolecular Glaser‐like coupling. b) Intramolecular Glaser‐like coupling. c) Synthesis of compound **1**.

Intermolecular coupling reactions are challenging to initiate by atom manipulation, in part because of the difficulty of aligning the precursor molecules. Performing such reactions intramolecularly, facilitates inducing the reaction, because the reacting functional groups can be pre‐aligned by design. Intramolecular reaction‐experiments can tell us if such coupling reactions as the Glaser reaction are in principle possible by atom manipulation and could reveal details about the reaction pathway and its intermediates. With this idea in mind we envisaged the synthesis of compound **1** as an ideal molecular model to explore this reaction by atom manipulation (Scheme 1 b). The expected proximity between the terminal alkynes would facilitate the C−C bond formation (in red) to obtain the 1,8‐anthrylene‐ethynylene cyclic dimer **2**.

Although compound **2** was not previously reported, the synthesis of an alkyl substituted derivate had been achieved by Toyota and co‐workers in 2007 by means of a double Sonogashira reaction in solution.^[23]^ For the synthesis of compound **1** we modified a procedure developed by this group, starting with commercially available 1,8‐dibromoanthracene **3** (Scheme 1 c).^[24]^ Double Sonogashira coupling between compound **3** and trimethylsilylacetylene (TMS=trimethylsilyl), followed by the fluoride‐induced deprotection of the alkynes and the selective introduction of one triisopropylsilyl (TIPS) group led to the isolation of diyne **4**. Then, an intermolecular Glaser‐like homocoupling followed by fluoride‐induced cleavage of the TIPS afforded compound **1** as a yellow solid.

The precursor molecules **1** were deposited via sublimation from a Si wafer onto a cold (*T*≈10 K) Cu(111) surface partially covered with (100) oriented bilayer NaCl islands. The experiments were carried out in a combined scanning tunneling microscope/atomic force microscope (STM/AFM) system equipped with a qPlus force sensor^[25]^ operating at *T=*5 K in frequency‐modulation mode.^[26]^ We used CO tip functionalization to improve the resolution for AFM^[27]^ and STM.^[28]^ AFM images were acquired at constant height, with the tip‐height offset Δ*z* applied to the tip‐sample distance with respect to the STM setpoint above the bare NaCl surface.

After deposition of **1** on the substrate at *T=*10 K, we found three different conformations of the molecules on NaCl(2ML)/Cu(111), as shown in the CO‐tip AFM images in Figure 1 b–d. The majority (55 %) of molecules are found in the conformation shown in Figure 1 b, that we assign to *trans*‐**1**. In this conformation the molecule is prochiral and both corresponding enantiomeric adsorbates are observed with about equal occurrences. The characteristic bright contrast that relates to the triple bonds[^8^, ^30^, ^31^] is observed in the AFM images. The similar brightness of the entire molecule and the symmetric AFM contrast indicate a planar adsorption geometry.^[32]^ A slightly smaller set of molecules (about 45 %) is found with the contrasts shown in Figure 1 c and d, that we assign to molecules adsorbed in the *cis* conformations (*P*)‐*cis*‐**1** and (*M*)‐*cis*‐**1**. The asymmetric contrast, i.e., the different brightness observed for the two anthracene moieties, indicates a non‐planar adsorption, explained by steric hindrance between the two terminal alkyne groups. This steric hindrance forces one terminal alkyne group being closer to the substrate (down) and the other one being further away (up), resulting in significantly brighter contrast above the latter. Thus, the *cis* conformation shows helicity and is chiral. Furthermore, the two anthracene moieties are not parallel to each other, probably also related to the steric hindrance between the end groups. These observations suggest that a carbon‐carbon bond between the alkyne end groups had not formed and does not form spontaneously at these cryogenic temperatures. As expected, we observed both enantiomers (*P*)‐*cis*‐**1** and (*M*)‐*cis*‐**1** with about equal occurrences.

**Figure 1:**
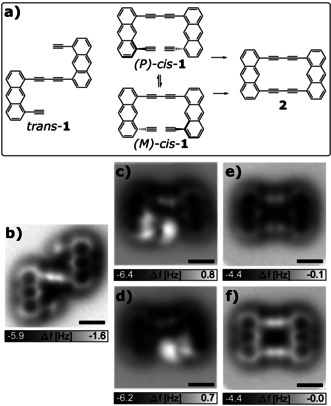
Precursors and product. a) Schemes of the precursor molecule **1** in the *trans* and *cis* conformations, and the product **2**. b) Constant‐height AFM image with a CO‐functionalized tip on a precursor in the *trans* conformation on bilayer NaCl on Cu(111), tip‐height offset Δ*z*=0.85 Å, i.e., the tip‐sample spacing was increased by 0.85 Å from a STM setpoint of *I*=1 pA and *V=*0.1 V. Panels c) and d) show two enantiomeric conformations of *cis*‐**1**, before and after its helicity was changed from (*P*) to (*M*) by atom manipulation, Δ*z*=1.0 Å in (c) and Δ*z*=1.1 Å in (d). Panels e) and f) show the product after a tip‐induced Glaser coupling reaction, imaged at tip‐height offsets of Δ*z*=1.7 Å in (e) and Δ*z*=1.5 Å in (f). The data shown in (e) and (f) were recorded with a different tip than (b)–(d). All scale bars are 5 Å.

It was possible to switch the helicity of compound *cis*‐**1**,[^33^, ^34^] i.e., between (*P*)‐*cis*‐**1** and (*M*)‐*cis*‐**1** in both directions. Such switching occurred frequently during AFM imaging when the tip height was decreased with respect to the tip height in Figure 1 c and d (see Supporting Information). We never observed a conformational change between the *trans*‐**1** and *cis*‐**1** conformations on the surface.

To study if Glaser‐like coupling reactions can be induced by atom manipulation we focused on the *cis*‐**1** conformers, in which the end groups are aligned and positioned to facilitate a possible coupling of the terminal alkynes. To induce the reaction, the tip was positioned above such a molecule on bilayer NaCl on Cu(111) and retracted by 9–11 Å from a tunneling setpoint of *V*=1 V and *I*=1 pA. Then the sample voltage was ramped up to sample voltages *V*>4.5 V, (*V*=5.6 V in the case shown in Figure 1. The pulse was applied for several seconds, the measured tunneling current *I* during the pulse was up to 400 pA. We did not observe dehydrogenation for pulses with voltages *V*<+4 V and we were not successful in inducing the Glaser coupling with pulses of negative sample polarity. We observed steps in the tunneling current during the voltage pulses; however, we cannot unambiguously differentiate steps caused by dehydrogenation events from those caused by a lateral displacement of the molecule or switching between *cis*‐**1** conformers. Usually the molecule was displaced during a voltage pulse that caused dehydrogenation and thus only AFM imaging, after such manipulation of a *cis*‐**1** precursor, revealed the result of the manipulation.

That way we created the molecule shown in Figure 1 e and f that we assign to the Glaser coupled product **2**. Our yield for generating **2** from *cis*‐**1** using the described voltage pulses was about 12 %. That is, 12 % of the *cis*‐**1** precursors that we attempted (in total N=34) were converted successfully into **2** by the described voltage pulses. The unsuccessful attempts resulted in products/fragments that could not be identified, or molecules being picked up by the tip.

We investigated the electronic structure of **2** by obtaining STM images at the negative ion resonances, which can be related to orbital density maps of the lowest unoccupied molecular orbitals (LUMOs) of the molecule.^[12]^ The positive ion resonance, related to the highest occupied molecular orbital, could not be experimentally accessed. In differential conductance (d*I*/d*V*) spectroscopy, we observed peaks centered at +1.5 V, +2.0 V and a third one with onset at +2.3 V, that we assign to resonant tunneling into the LUMO, LUMO+1 and LUMO+2, respectively (see Supporting Information). We obtained STM constant‐current images at these voltages using a CO terminated tip, i.e., a tip with a partial *p*‐wave character,^[28]^ see Figure 2 first row, and a Cu tip, i.e., an *s*‐wave tip, see Figure 2 second row.

**Figure 2:**
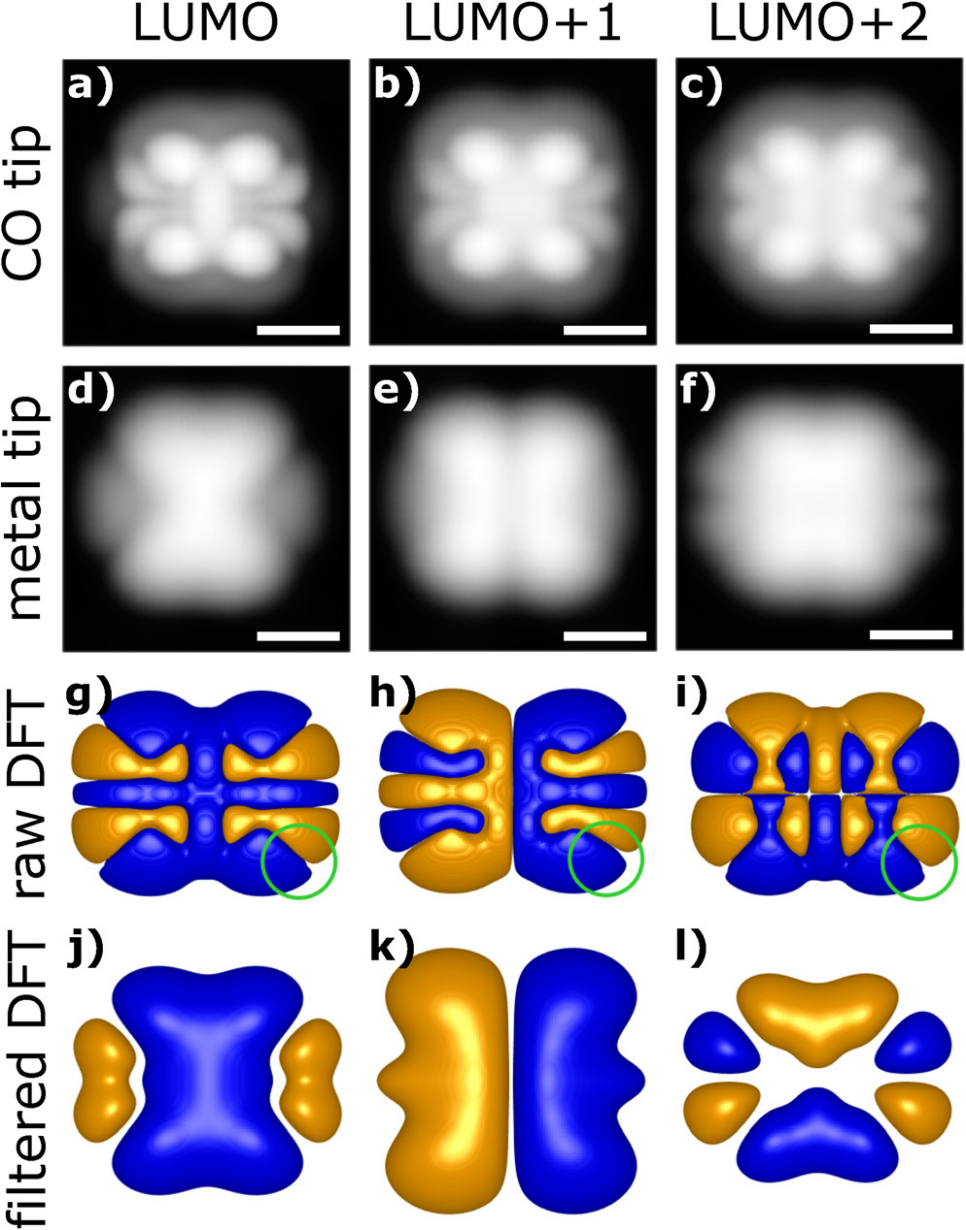
Orbital densities of reaction product. STM orbital‐density maps and calculations of the LUMO, LUMO+1, and LUMO+2 densities of **2**. STM constant‐current images recorded at a current of 1 pA with a CO‐functionalized tip (bias voltages of a) 1.5 V, b) 2.0 V, and c) 2.5 V) and with a Cu tip (bias voltages of d) 1.5 V, e) 2.1 V, and f) 2.5 V) on **2** adsorbed on NaCl(2ML)/Cu(111). Black to white contrast corresponds to tip‐height ranges of 3.5 Å for panels a) and f) and of 4.0 Å for panels b) to e). All scale bars correspond to 5 Å. g–i) The corresponding iso‐surfaces of calculated orbital wavefunctions. j–l) Iso‐surfaces of DFT‐calculated orbitals after a 3D Gaussian filter was applied to mimic the spatial extension of the metallic tip. The green circles in (g–i) indicate the full width half maximum of this 3D Gaussian filter.

Additionally, we carried out density functional theory (DFT) calculations shown in Figure 2, third row. The orbitals of **2** can be rationalized as those of two coupled diethynylanthracene molecules, with the LUMO and LUMO+1 of **2** displaying a bonding and an antibonding combination^[35]^ of two diethynylanthracene LUMOs, respectively and the LUMO+2 of **2** displaying a bonding combination of two diethynylanthracene LUMO+1 orbitals (see Supporting Information).

To compare with the images obtained with the Cu (*s*‐wave) tip, we folded the calculated wavefunction with a spherical function to account for the *s*‐wave character and finite size of the tip,[^35^, ^36^] see Figure 2 fourth row. The agreement of the calculated wavefunctions with the measured orbital densities (corresponding to wavefunctions squared) is excellent, confirming the creation of **2** by atom manipulation. Compared to the Cu tip, the images of the CO tip show larger contrast and corrugation due to the larger tunneling resistance, resulting in a smaller tunneling gap^[12]^ and due to the partial *p*‐wave character of the CO tip.^[28]^ We mainly assign the differences between the CO tip images and the unfiltered (raw) DFT results to the partial *p*‐wave character of the CO tip.[^28^, ^29^]

A theoretical study of the reaction mechanism of the thermally induced Glaser‐like coupling on Ag(111) indicated that the carbon‐carbon bond is formed first and then dehydrogenation occurs, with the latter being the limiting step of the reaction.^[37]^ Such intermediates, after carbon‐carbon bond formation but before dehydrogenation have been observed in on‐surface cyclodehydrogenation^[38]^ but not in Glaser‐like coupling reactions. After performing voltage pulses above *cis*‐**1** we once observed reaction intermediates **5** and **6**, both with an additional hydrogen atom (with respect to **2**) still attached to the molecule, see Figure 3 b and c, respectively. This observation suggests that carbon‐carbon bond formation occurred before dehydrogenation of the second hydrogen atom (in red in Figure 3 a). It also suggests that the second hydrogen, which still needs to be dissociated from **2**, is relatively mobile within the molecule. Supporting the latter point, i.e., the possible conversion between **5** and **6**, we observed that the position of the additional hydrogen could be altered by atom manipulation. A voltage pulse (*V*=4 V) resulted in a change of the position of the additional hydrogen from the anthracene moiety in **5,** see Figure 3 b, to the acetylene bridge in **6**, see Figure 3 c. When we applied a voltage pulse of 4.5 V above **6** in Figure 3 c, it resulted in dehydrogenation and completed the synthesis of **2**, see Figure 3 d. From our observations we cannot infer whether the first hydrogen dissociation occurred before or after C−C bond formation.

**Figure 3:**
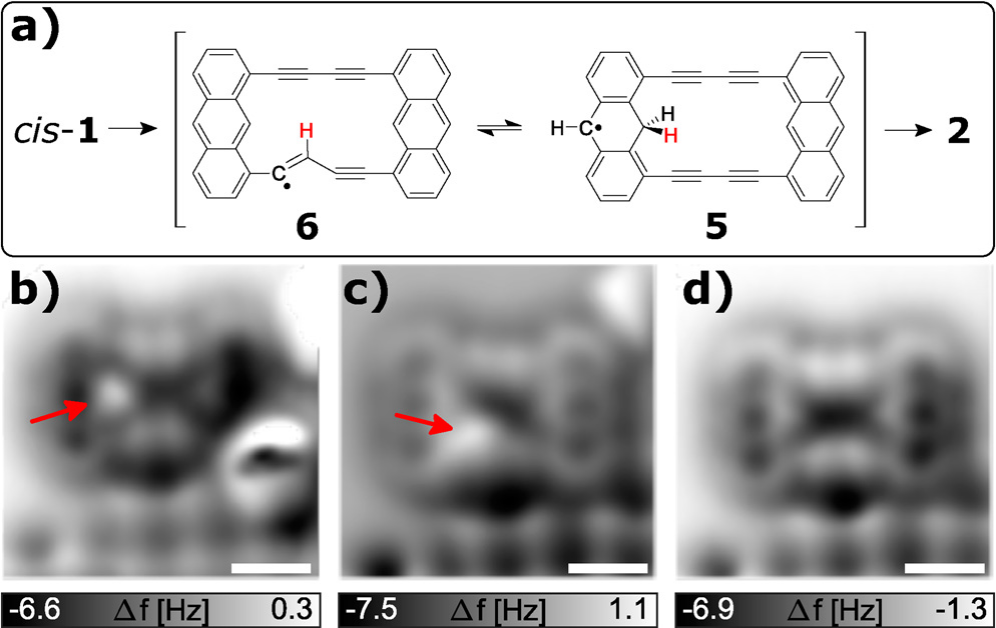
Reaction intermediates. a) Reaction scheme with observed intermediates. b–d) Constant‐height AFM with CO tip. The molecule was anchored next to a third layer patch of NaCl (bottom of images). Red arrows indicate the position of the additional hydrogen atoms. Between recording (b), assigned as **5**, and (c), assigned as **6**, a voltage pulse of 4 V was applied. Between recording (c) and (d), assigned as **2**, a voltage pulse of 4.5 V was applied. Constant‐height AFM with CO tip and tip‐height offsets Δ*z*=1.4 Å in (a), 1.0 Å in (b) and 1.1 Å in (c) with respect to the STM setpoint of 1 pA and 0.1 V. All scale bars correspond to 5 Å.

Our results demonstrate that Glaser‐like coupling reactions can be induced by atom manipulation and revealed intermediates indicating that the second dehydrogenation step occurred after carbon‐carbon bond formation. This work shows that the proper design of the molecular precursor can be crucial to facilitate on‐surface reactions by atom manipulation, providing unprecedented insights into the reaction mechanisms in single molecules.

## Conflict of interest

The authors declare no conflict of interest.

## Supporting information

As a service to our authors and readers, this journal provides supporting information supplied by the authors. Such materials are peer reviewed and may be re‐organized for online delivery, but are not copy‐edited or typeset. Technical support issues arising from supporting information (other than missing files) should be addressed to the authors.

Supplementary
