# Supplementary material for: Intramolecular Coupling of Terminal Alkynes by Atom Manipulation
Source: Angew Chem Int Ed Engl. 2020 Oct 13;59(51):22989–93. doi: 10.1002/anie.202009200 (PMC7756451; doi:10.1002/anie.202009200)
Supplement: Supplementary file 1 — Supplementary [file ANIE-59-22989-s001.pdf]

Angewandte Chemie
Eine Zeitschrift der Gesellschaft Deutscher Chemiker

Supporting Information

Intramolecular Coupling of Terminal Alkynes by Atom Manipulation

Florian Albrecht[+], Dulce Rey[+], Shadi Fatayer, Fabian Schulz, Dolores Pérez, Diego Peña,* and Leo Gross**

anie_202009200_sm_miscellaneous_information.pdf

Synthesis of precursor molecules 1:

General methods for the solution synthesis

All reactions were carried out under argon using oven-dried glassware. *n*-BuLi was used in solution in hexane (2.4 M). TBAF was used in solution in THF (1 M). Et_3N, *i*-Pr_2NH and *i*-Pr_3SiCl were dried by distillation over CaH_2. Commercial reagents were purchased from ABCR GmbH, Sigma-Aldrich or TCI Chemicals and were used without further purification. THF and CH_2Cl_2 were purified by a MBraun SPS-800 Solvent Purification System. TLC was performed on Merck silica gel 60 F_{254} and chromatograms were visualized with UV light (254 and 365 nm). Flash column chromatography was performed on Merck silica gel 60 (ASTM 230-400 mesh). ^1H and ^{13}C NMR spectra were recorded at 300 and 75 MHz, respectively (Varian Mercury 300). APCI spectra were determined on a Bruker Microtof instrument. Deuterated solvents were purchased from Acros Organics.

Preparation of 1,8-bis((trimethylsilyl)ethynyl)anthracene (7)

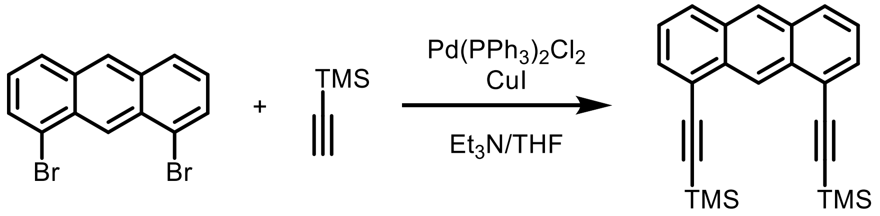

3 **7**

Figure S1. Synthesis of **7**

Dibromanthracene **3** (250 mg, 1.0 mmol), $Pd(PPh)_3)_2Cl_2$ (53 mg, 0.075 mmol) and CuI (8 mg, 0.075 mmol) were dissolved in a mixture of Et_3N/THF (1:1, 15 mL). Trimethylsilylacetylene (260 µL, 2.5 mmol) was added and the reaction mixture was stirred at 90 ºC for 24 h. Then, the solvent was evaporated under reduced pressure and the residue purified by column chromatography (SiO_2; hexane/CH_2Cl_2, 10:1), to isolate compound **7** (221 mg, 80%) as a yellowish solid. [1]

^{1}H NMR (300 MHz, $CDCl_3$) δ: 9.33 (s, 1H), 8.40 (s, 1H), 7.97 (d, *J* = 8.7 Hz, 2H), 7.79 (d, *J* = 6.3 Hz, 2H), 7.41 (dd, *J* = 8.7, 6.6 Hz, 2H), 0.40 (s, 18H) ppm.

Preparation of 1,8-diethynylanthracene (8)

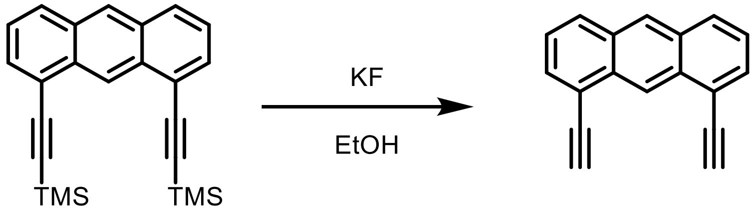

Figure S2. Synthesis of **8**

Compound **7** (275 mg, 0.74 mmol) and KF (215 mg, 3.74 mmol) were dissolved in ethanol (18 mL) and the reaction mixture was reflux for 2 h. Then, the solvent was evaporated under reduced pressure and the residue purified by column chromatography (SiO_2; hexane), to isolate diyne **8** (135 mg, 80%) as a white solid. [2]

^{1}H NMR (300 MHz, $CDCl_3$) δ: 9.43 (s, 1H), 8.41 (s, 1H), 7.99 (d, *J* = 8.4 Hz, 2H), 7.79 (d, *J* = 6.9 Hz, 2H), 7.43 (dd, *J* = 8.4 Hz y 6.9 Hz, 2H), 3.63 (s, 2H) ppm.

Preparation of ((8-ethynylanthracen-1-yl)ethynyl)triisopropylsilane (4)

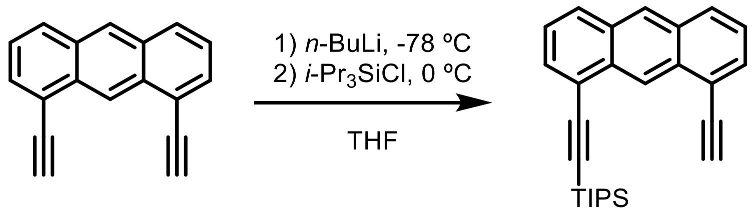

Figure S3. Synthesis of **4**

n-BuLi (196 µL, 0.49 mmol, 2.5 M) was added dropwise to a solution of diyne **8** (100 mg, 0.44 mmol) in THF (5 mL) at -78 ºC and the mixture was stirred for 1.5 h at this temperature. Then, *i*-Pr_3SiCl (95 µL, 0.44 mmol) was added dropwise at 0 ºC, the mixture was stirred for 3 h at 0 ºC and allowed to warm up to room temperature for 16 h. Then H_2O was added, the organic phase was separated and the aqueous phase was extracted with CH_2Cl_2 (3 x 3 mL). The combined organic phases were dried over Mg_2SO_4. The solvent was evaporated under reduced pressure and the residue was purified by column chromatography (SiO_2; hexane), to afford **4** (140 mg, 83 %) as a yellow oil. [2]

^{1}H NMR (300 MHz, $CDCl_3$) δ: 9.64 (s, 1H), 8.45 (s, 1H), 8.03 (d, *J* = 8.6 Hz, 1H), 8.00 (d, *J* = 8.6 Hz, 1H), 7.79 (d, *J* = 7.1 Hz, 2H), 7.44 (dd, *J* = 7.1, 8.6 Hz, 2H), 3.49 (s. 1H), 1.16-1.24 (21H, m) ppm.

Preparation of 1,4-bis(8-((triisopropylsilyl)ethynyl)anthracen-1-yl)buta-1,3-diyne (9)

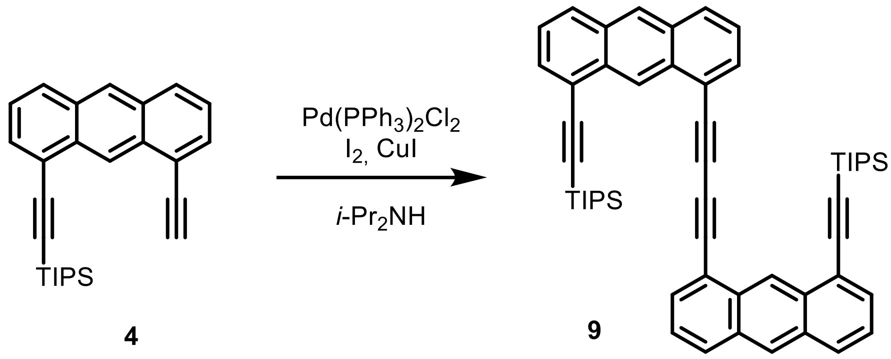

Figure S4. Synthesis of **9**

Compound **4** (230 mg, 0.60 mmol) was disolved in *i*-Pr_2NH (6 mL). Then, $Pd(PPh_3)_2Cl_2$ (21.0 mg, 30.0 µmol), CuI (12.0 mg, 60.0 µmol) and I_2 (76 mg, 30 µmol) were added and the reaction mixture was stirred at room temperature for 16 h. The solvent was evaporated under reduced pressure and the residue purified by column chromatography (SiO_2, hexane/CH_3Cl, 6:1) to afford compound **9** (80 mg, 35 %) as a yellow solid. [3]

^{1}H NMR (300 MHz, $CDCl_3$) δ: 9.50 (s, 2H), 8.46 (s, 2H), 8.05 (d, *J* = 8.3 Hz, 2H), 7.99 (d, *J* = 8.3 Hz, 2H), 7.89 (d, *J* = 6.3 Hz, 2H), 7.78 (d, *J* = 6.3 Hz, 2H), 7.48 (dd, *J* = 6.8, 8.3 Hz, 2H), 7.44 (dd, *J* = 6.8, 8.3 Hz, 2H), 1.12 (septet, *J* = 6.3 Hz, 6H), 1.06 (d, *J* = 6:3 Hz, 36H) ppm.

Preparation of 1,4-bis(8-ethynylanthracen-1-yl)buta-1,3-diyne (1)

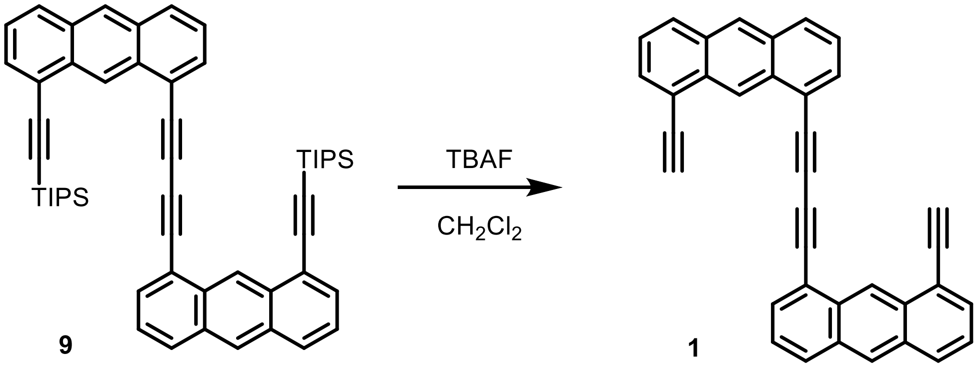

Figure S5. Synthesis of **1**

Over a solution of **9** (70 mg, 0.090 mmol) in CH_2Cl_2 (18 mL), TBAF (185 µL, 0.185 mmol, 1.0 M in THF) was added dropwise and the mixture was stirred at room temperature for 16 h. Then, H_2O (3 mL) was added, the organic phase was separated and the aqueous phase was extracted with CH_2Cl_2 (3 x 5 mL). The combined organic phases were dried over Mg_2SO_4. The solvent was evaporated under reduced pressure and the residue was purified by column chromatography (SiO_2, hexane/CH_3Cl, 7:1), to isolate compound **1** (25 mg, 61 %) as a yellow solid.

^{1}H NMR (300 MHz, $CDCl_3$) δ: 9.53 (s, 2H), 8.49 (s, 2H), 8.06 (dd, *J* = 8.7, 7.1 Hz, 4H), 7.92 (dd, *J* = 7.0, 1.1 Hz, 2H), 7.81 (dd, *J* = 6.9, 1.1 Hz, 2H), 7.53 – 7.44 (m, 4H), 3.70 (s, 2H) ppm.

^{13}C NMR (75 MHz, $CDCl_3$) δ: 132.62, 131.74, 131.57, 131.43, 131.31, 129.94, 129.41, 127.78, 125.13, 123.79, 120.46, 120.24, 83.44, 81.45, 79.57 ppm.

MS (APCI (M+1)) HR calculated for $C_{36}H_{19}$: 451.1481, found: 451.1482.

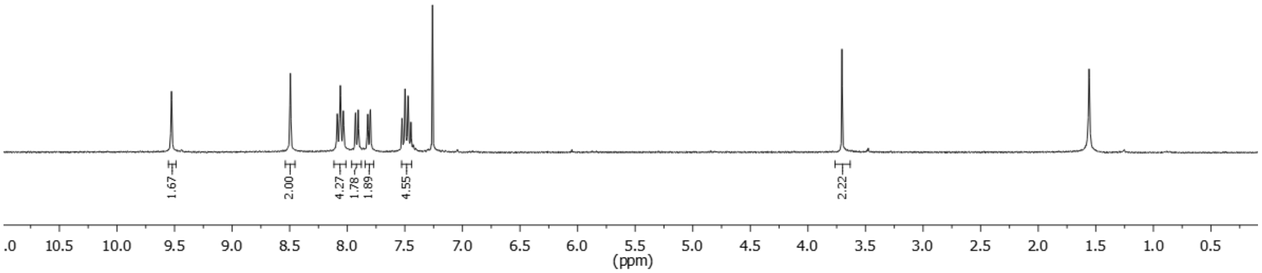

Figure S6. ^{1}H NMR spectra for **1**

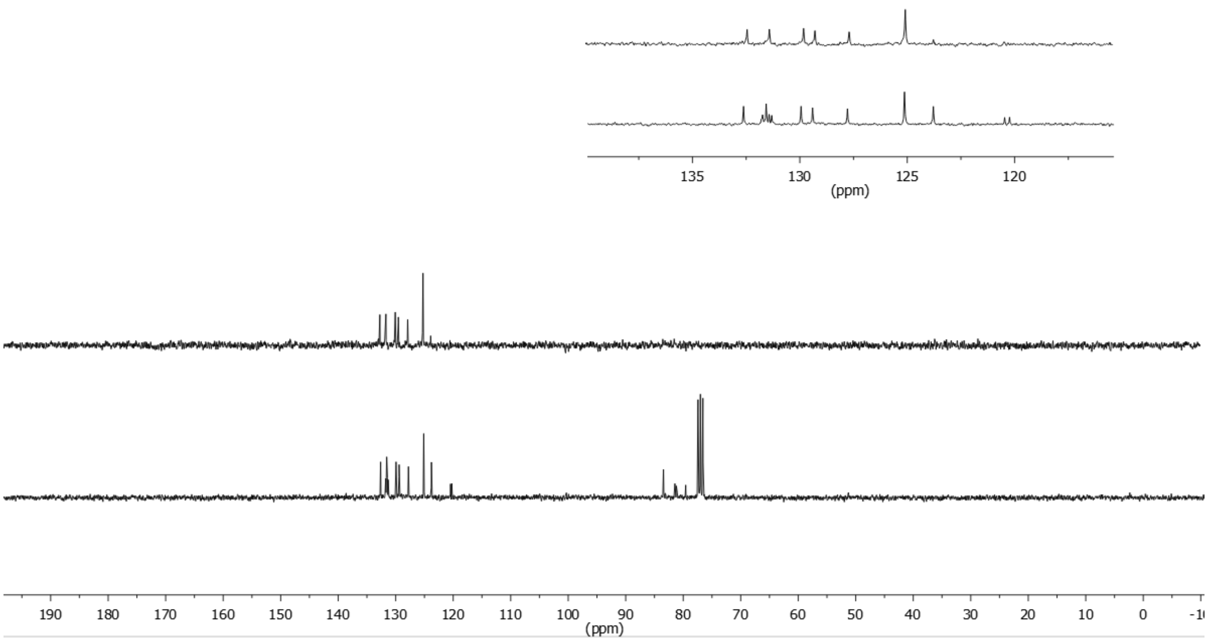

Figure S7. ^{13}C NMR spectra for **1**

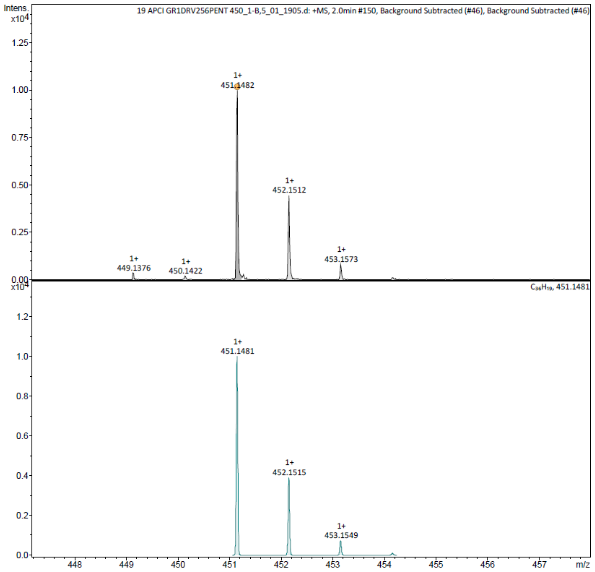

Figure S8. HRMS (APCI) for **1** (M+1).

Details of DFT simulations:

Density functional theory (DFT) simulations were performed using the FHI-AIMS code [4]. The structures of the reaction products as well as precursor *trans*-**1** were optimized in the gas phase. To mimic the molecule-substrate interaction effects onto the helical structure of the *cis*-**1** reactant, we relaxed this molecule on a bilayer NaCl slab of 8 x 8 atoms – consisting of 128 substrate atoms in total. The atom positions for the lower NaCl layer were constrained. All the simulations used the Perdew-Burke-Ernzerhof exchange-correlation functional [5].

Additional AFM and STM measurements:

Fig. S9 shows helicity switching of *cis*-**1** observed with the AFM

Figure S10 shows a tip induced Glaser coupling reaction, where the molecule changed its adsorption site only a little, because it was adsorbed at a step edge to a third layer NaCl patch.

Figure S11 shows I/V and d*I*/d*V* scanning tunneling spectroscopy above the reaction product **2**.

Figure S12 and S13 show measured orbital densities of the precursors *cis*-**1** and *trans*-**1**, respectively. DFT calculated orbital densities are shown in addition.

Figure S14 shows the calculated LUMO, LUMO+1 and LUMO+2 of **2** and in comparison, those of diethynylanthracene.

Figure S15 shows measured AFM images of **5** recorded at different tip-height offsets.

Figure S16 shows simulated AFM images of all assigned molecular structures.

Figure S17 shows experimental images of **1** adsorbed on clean Cu(111).

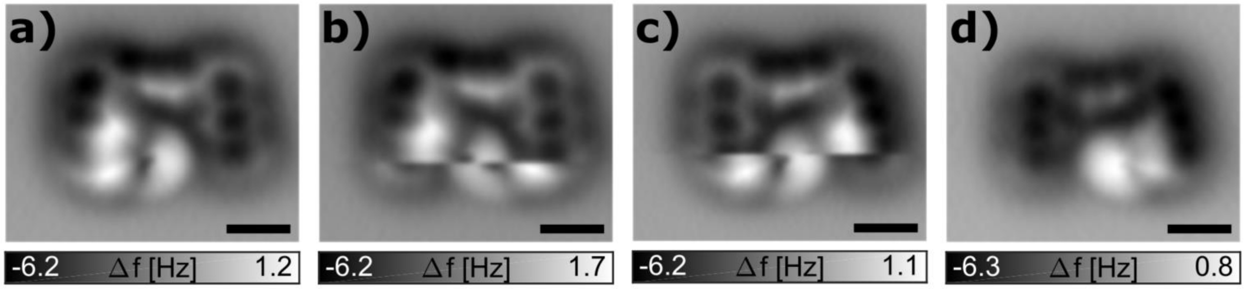

Fig S9: Helicity switching of *cis*-1 while imaging. a)-d) series of AFM images on the same individual molecule. a) and d) show the molecule in *(P)-cis*-**1** and *(M)-cis*-**1** conformation, respectively. b) and c) show AFM images during which the helicity was switched. The helicity of the molecule switched from *(P)-cis-**1*** to *(M)-cis-**1*** in the lower part of b). The molecule switched back from *(M)-cis-**1*** to *(P)-cis-**1*** in c). The slow scan direction was always from top to bottom. The images were recorded at tip-height offsets Δ*z* = 0.9 Å, 0.8 Å, 0.9 Å and 1.0 Å for panels a), b), c) and d), respectively, with respect to the STM setpoint of *I* = 1 pA at *V* = 0.1 V on bare NaCl. All scale bars correspond to 5 Å.

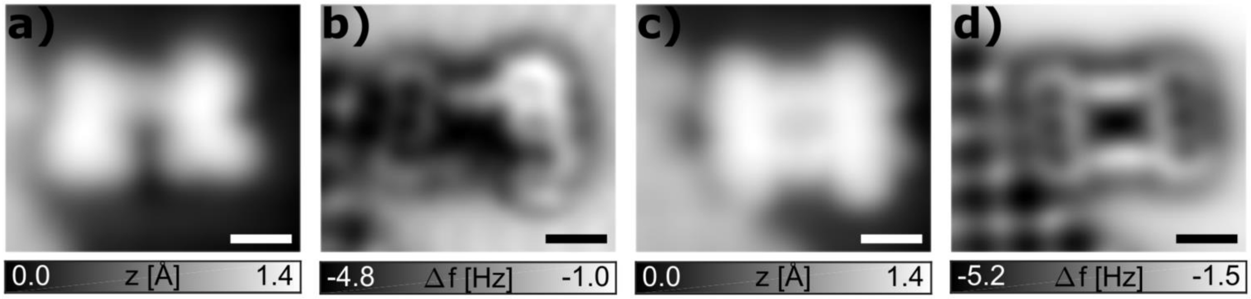

Fig S10: Imaging of precursor *cis*-1 and product 2 adsorbed next to a patch of third layer NaCl, before and after the tip-induced coupling reaction. a) shows a constant-current STM map and b) a constant-height AFM image (Δ*z* = -0.10 Å) of precursor *cis*-**1**. c) and d) show STM and AFM measurements after applying a voltage pulse of 5.75 V, generating **2**. Parameters: a) and c) *I* = 1 pA, *V* = 1.25 V; b) and d) Δ*z* = -0.10 Å and Δ*z* = -0.15 Å, respectively, with respect to the STM setpoint of *I* = 1 pA, *V* = 1.25 V.

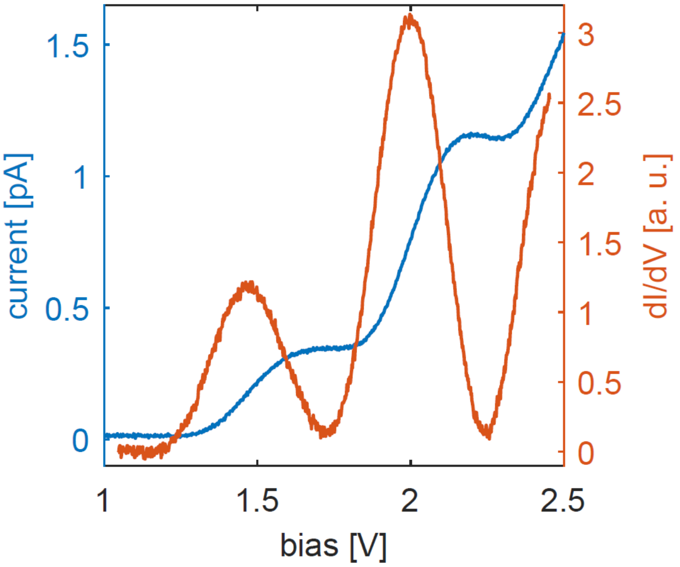

Fig S11: Differential conductance spectrum of reaction product 2. The blue curve shows the tunneling current *I* recorded with a metal tip over the reaction product **2** as a function of sample bias voltage *V*. The red curve shows the numerically derived differential conductance curve (d*I*/d*V*). The peaks at 1.5 and 2.0 V are assigned to the LUMO and the LUMO+1 of the product **2**. The third feature, with onset at 2.3 *V*, is assigned to the LUMO+2.

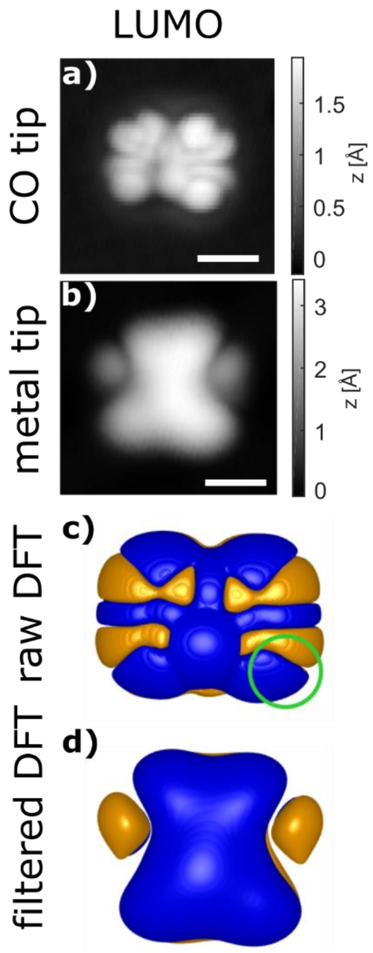

Fig S12: Orbital density imaging and simulated orbitals of *(M)-cis*-1. Constant-current STM images of the LUMO densities of *(M)-cis*-**1** adsorbed on bilayer NaCl with a) CO terminated tip and b) Cu terminated tip. Parameters: a) I = 1 pA, V = 1.5 V; b) I = 1 pA, V = 1.6 V. c) unprocessed DFT derived LUMO iso-surface and d) iso-surface of LUMO after applying a filter as indicated by the green circle in c). The molecule is oriented similar to Fig. 2d) of the main text.

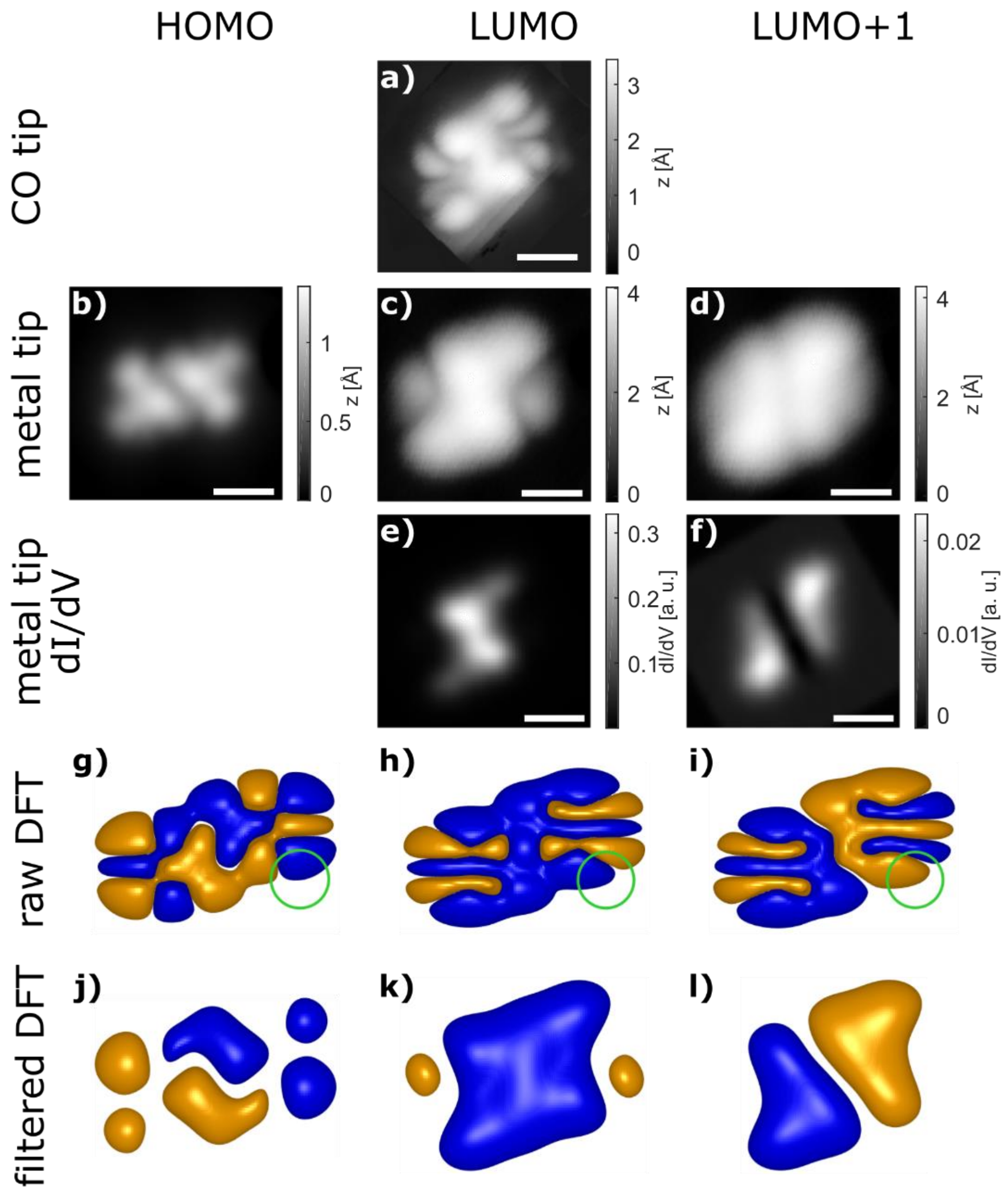

Fig S13: Orbital density imaging and simulated orbitals of *trans*-1. a)-f) show experimental orbital density imaging of the molecule adsorbed on bilayer NaCl with a) CO tip and b)-f) Cu tip. Parameters: a) I = 1 pA, V = 1.5 V; b) I = 2 pA, V = -2.8 V; c) I = 1 pA, V = 1.5 V; d) I = 1 pA, V = 2.1 V. e), f) dI/dV images acquired at constant height with a sinusoidal modulation at 163 Hz and V_{ac} = 50 mV applied in addition to the dc voltage V_{dc} to extract the dI/dV signal using lock-in technique. Parameters: e) Setpoint I = 10 pA, V = 1.5 V, Δz = 2.5 Å, V_{dc} = 1.5; f) Setpoint I = 10 pA, V = 2.0 V, Δz = 4.0 Å, V_{dc} = 2.0 V. g) – i) unprocessed DFT derived iso-surfaces of orbitals and j) – l) iso-surfaces of orbitals after applying a filter as indicated by the green circle in g)-i). The molecule is oriented similarly to Fig. 2b) of the main text.

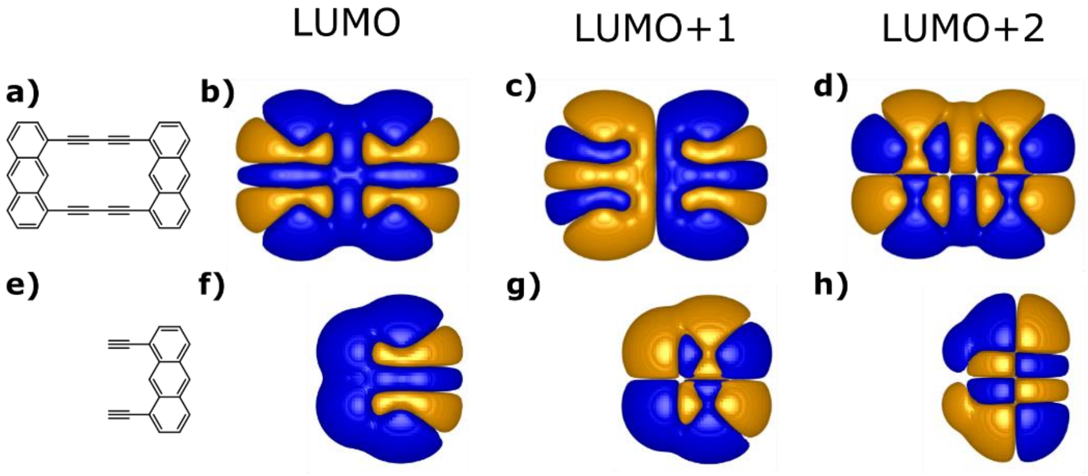

Fig S14: DFT derived orbitals of 2 and diethynylanthracene. b)-d) show the three lowest unoccupied orbitals of **2** and f)-h) those of diethynylanthracene. The models a) and e) provide the orientation of both molecules. Combining two LUMOs of diethynylanthracene in a bonding (symmetric) and antibonding (antisymmetric) manner rationalize the LUMO and LUMO+1 of **2**, respectively. The bonding combination of two LUMO+1 orbitals of diethynylanthracene rationalizes the LUMO+2 of **2.**

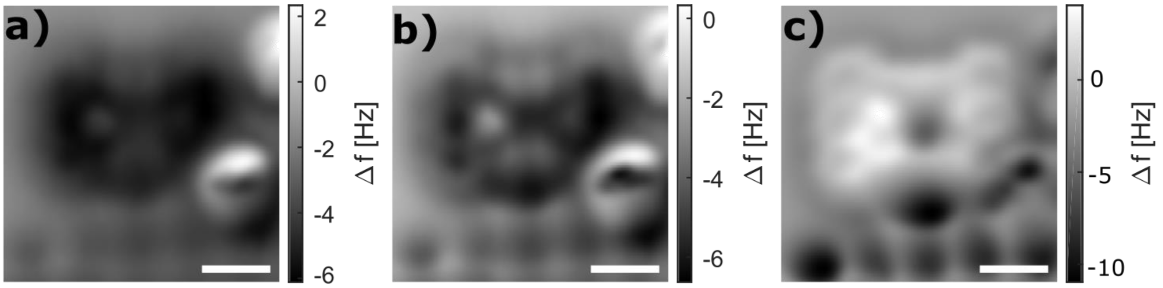

Fig S15: AFM images of 5 recorded at different tip-height offsets. Same molecule as in the main text Fig. 3b) recorded at tip-height offsets of 1.6 Å (a)), 1.4 Å (b)) and 0.8 Å (c)). Setpoint I = 1 pA, V = 0.1 V. The two features at the right-hand side of the images (bright in a) and b)), are assigned to individual CO molecules, adsorbed nearby **5**. All scale bars correspond to 5 Å.

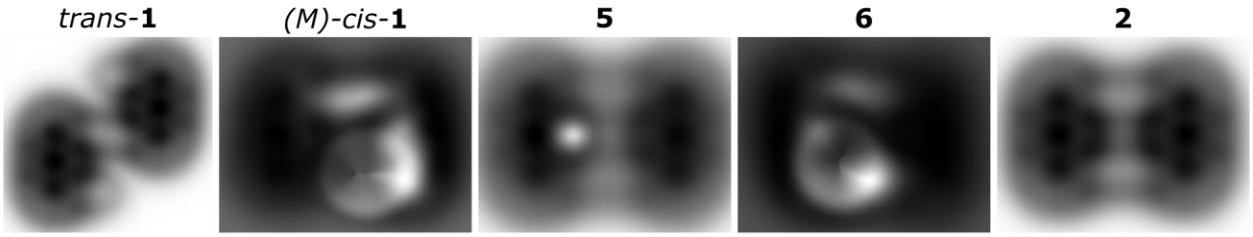

Fig S16: Simulated AFM images of all assigned molecular structures. The atomic positions for the molecules are calculated using DFT and relaxed in the gas phase, except for the *(M)-cis*-1 which is adsorbed on bilayer NaCl (for further methods, see the details of DFT simulations on page 6 of the SI). The orientations of the molecules correspond to the models shown in Fig. 1a) and 3a) of the main text. The AFM simulations were done using the probe particle model [6] with a tip charge of -0.05 electrons and a lateral spring constant of the CO molecule at the tip of 0.2 N/m.

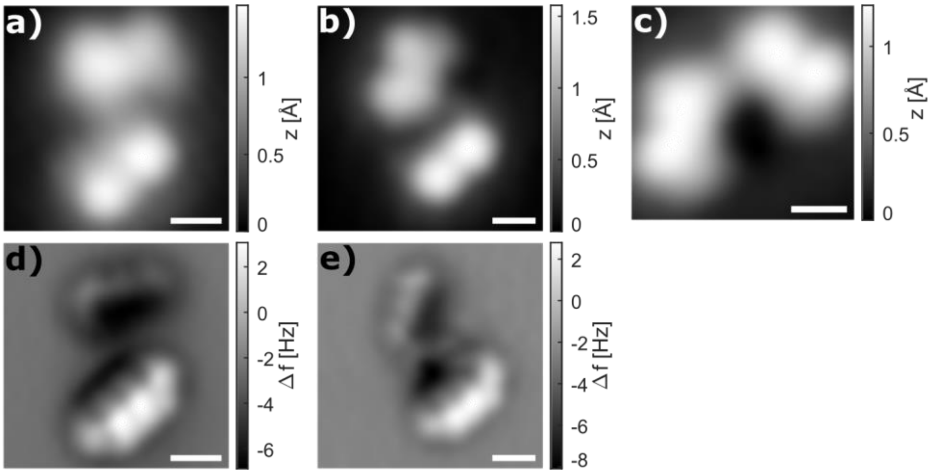

Fig S17: Precursor molecules adsorbed on clean Cu(111). Panels a)-c) show constant current STM images and d)-e) corresponding constant-height AFM images. Due to the interaction with the metallic substrate the molecules are distorted. The long axes of the anthracene moieties are not parallel and both anthracene moieties are tilted with respect to the surface plane. Images were recorded at I = 5 pA and V = 0.3 V for panel a), I = 5 pA and V = 0.1 V for b) and c). The tip-height offsets where -1.4 Å (with respect to the setpoint of I = 5 pA and V = 0.3 V) for d) and -1.0 Å (from the setpoint of I = 5 pA and V = 0.1 V) for e). All scale bars correspond to 5 Å.

References

[1] F. Vögtle, H. Koch, K. Rissanen, Tweezer-Shaped Hydrocarbons, *Chem. Ber*. **1992**, *125*, 2129-2135.

[2] S. Toyota, M. Goichi, M. Kotani, M. Takezaki, Chemistry of Anthracene–Acetylene Oligomers. II. Synthesis, Structure, and Properties of 1,8-Anthrylene–Ethynylene Cyclic Tetramers and Related Acyclic Oligomers, *Bull. Chem. Soc. Jpn*. **2005**, *78*, 2214-2227.

[3] S. Toyota, H. Miyahara, M. Goichi, K. Wakamatsu, T. Iwanaga, Chemistry of Anthracene–Acetylene Oligomers. X. Synthesis, Structures, and Properties of 1,8-Anthrylene–Alkynylene Cyclic Trimers, *Bull. Chem. Soc. Jpn*. **2008**, *81*, 1147-1157.

[4] V. Blum, R. Gehrke, F. Hanke, P. Havu, V. Havu, X. Ren, K. Reuter, M. Scheffler, *Ab initio* molecular simulations with numeric atom-centered orbitals, *Comput. Phys. Commun.* **180**, 2175–2196 (2009).

[5] J. P. Perdew, K. Burke, M. Ernzerhof, Generalized gradient approximation made simple, *Phys. Rev. Lett.* **77**, 3865 (1996).

[6] P. Hapala, G. Kichin, C. Wagner, S. F. Tautz, R. Temirov, P. Jelínek, Mechanism of high-resolution STM/AFM imaging with functionalized tips, *Phys. Rev. B* **90**, 085421 (2014)
